# The interplay between m6A modification and non-coding RNA in cancer stemness modulation: mechanisms, signaling pathways, and clinical implications

**DOI:** 10.7150/ijbs.60641

**Published:** 2021-06-26

**Authors:** Sha Qin, Yitao Mao, Haofan Wang, Yingxing Duan, Luqing Zhao

**Affiliations:** 1 Department of Pathology, Xiangya Hospital, Central South University, Changsha, Hunan, China; and Department of Pathology, School of Basic Medical Science, Xiangya School of Medicine, Central South University, Changsha, Hunan, China.; 2 Department of Radiology, Xiangya Hospital, Central South University, Changsha, Hunan, China.; 3 National Clinical Research Center for Geriatric Disorders, Xiangya Hospital, Central South University, Changsha, Hunan, China.; 4 Department of Interventional Radiology, The 3rd Affiliated Hospital of Sun Yat-Sen University, Guangzhou, Guangdong, China.

**Keywords:** N6-methyladenosine (m6A) modification, non-coding RNA (ncRNA), cancer stemness, signaling pathways, biomarkers

## Abstract

Cancer stemness, mainly consisting of chemo-resistance, radio-resistance, tumorigenesis, metastasis, tumor self-renewal, cancer metabolism reprogramming, and tumor immuno-microenvironment remodeling, play crucial roles in the cancer progression process and has become the hotspot of cancer research field in recent years. Nowadays, the exact molecular mechanisms of cancer stemness have not been fully understood. Extensive studies have recently implicated that non-coding RNA (ncRNA) plays vital roles in modulating cancer stemness. Notably, N6-methyladenosine (m6A) modification is of crucial importance for RNAs to exert their biological functions, including RNA splicing, stability, translation, degradation, and export. Emerging evidence has revealed that m6A modification can govern the expressions and functions of ncRNAs, consequently controlling cancer stemness properties. However, the interaction mechanisms between ncRNAs and m6A modification in cancer stemness modulation are rarely investigated. In this review, we elucidate the recent findings on the relationships of m6A modification, ncRNAs, and cancer stemness. We also focus on some key signaling pathways such as Wnt/β-catenin signaling, MAPK signaling, Hippo signaling, and JAK/STAT3 signaling to illustrate the underlying interplay mechanisms between m6A modification and ncRNAs in cancer stemness. In particular, we briefly highlight the clinical potential of ncRNAs and m6A modifiers as promising biomarkers and therapeutic targets for indicating cancer stemness properties and improving the diagnostic precision for a wide variety of cancers.

## Introduction

**Figure 1:**
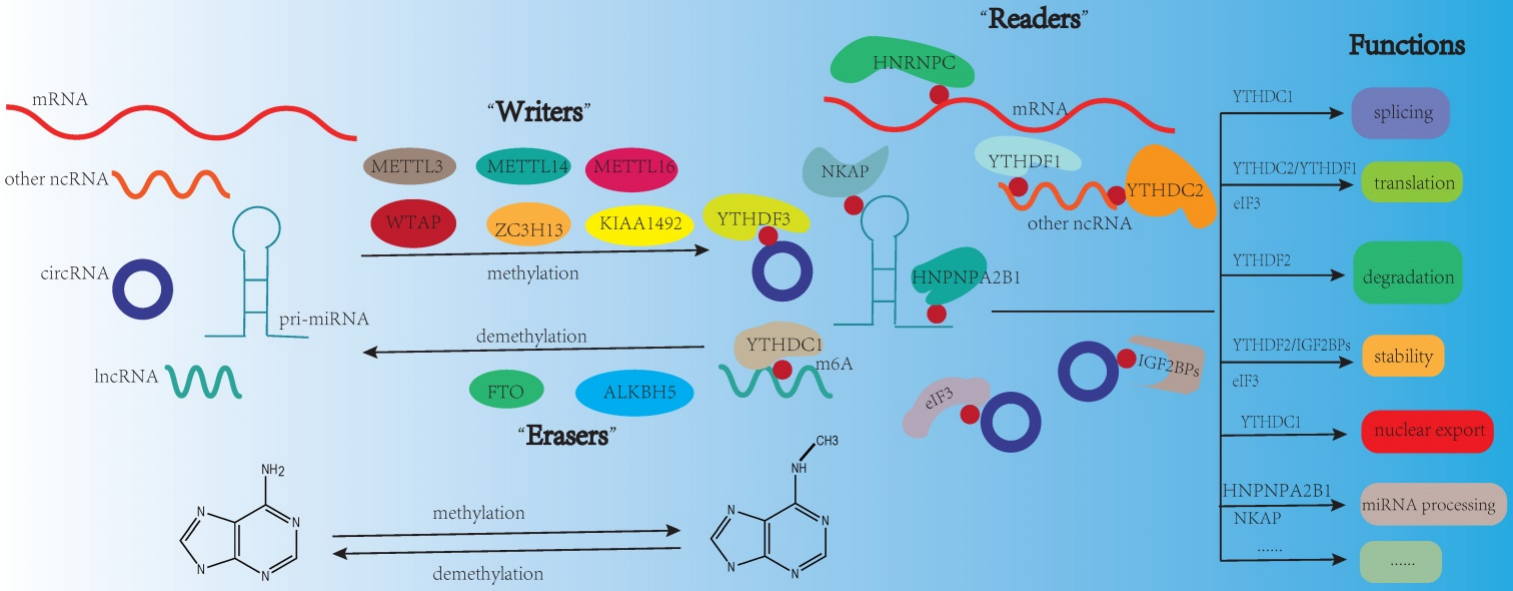
m6A modification on RNA. m6A modification is dynamic and reversible. m6A “writers” (METTL3, METTL14, METTL16, WTAP, ZC3H13, KIAA1492) could catalyze the m6A modification of adenosine on RNA. m6A “erasers” mainly include FTO and ALKBH5. These proteins can remove the methylation from RNA. m6A “readers” (YTHDF3, YTHDC1, YTHDC2, HNRNPC, HNPNPA2B1, YTHDF1, IGF2BPs, NKAP, eIF3) are selective RNA-binding proteins that can recognize m6A modification. The functions of m6A modification on RNA mainly include splicing, translation, degradation, stability, nuclear export, and miRNA processing.

N6-methyladenosine (m6A), the most prevalent modification in eukaryotic mRNAs, regulates RNA stability, splicing, degradation, translation, and export 1. The functions of m6A modification are governed by three homologous factors including “writers”, “readers” and “erasers”. m6A “writers” could catalyze the m6A modification of adenosine on RNA, which mainly including methyltransferase-like 3 protein (METTL3), methyltransferase-like 14 protein (METTL14), and WT1 associated protein (WTAP). m6A “writers” also contain RNA-binding motif protein 15/15B (RBM15/15B), methyltransferase-like 16 protein (METTL16), zinc finger CCCH-type containing 13 (ZC3H13), and Vir-like m6A methyltransferase associated (VIRMA, also known KIAA1429). m6A “erasers” mainly include FTO and ALKBH5. These proteins can remove the methylation from RNA, which means m6A modification is dynamic and reversible. m6A “readers” are selective RNA-binding proteins that can recognize m6A, which mainly include the YT521-B homology (YTH) domain-containing protein family (including YTHDFs like YTHDF1/2/3, and YTHDCs like YTHDC1/2). As a nuclear m6A “reader”, YTHDC1 is related to mRNA splicing and nuclear export 2, 3. YTHDC2 can positively regulate translation elongation through the interaction with RNA helicase 4. The functions of YTHDFs mainly depend on YTHDF1/2. YTHDF1 is linked to translation and metabolism 5, 6, while YTHDF2 is a well-studied m6A “reader” protein and influences mRNA stability and accelerates mRNA degradation 7. Insulin-like growth factor-2 mRNA-binding proteins (IGF2BPs including IGF2BP1/2/3) are another category of “reader”, they could promote the stability and storage of their target mRNAs to affect gene expression 8. In addition, the heterogeneous nuclear ribonucleoprotein (HNRNP) family (including HNRNPA2B1, HNRNPC and HNRNPG), NF-κB-associated protein (NKAP) and eukaryotic translation initiation factor 3 (eIF3) also belong to m6A “reader” 9 (**Fig. 1**). It is reported that m6A modification is enriched in the consensus sequence RRACH (where R: A or G and H: A, C, or U) and in 3′ untranslated regions (3′-UTRs), stop codons, as well as, internal long exons 10, 11.

Non-coding RNA usually lacks the potential to encode proteins, which contains microRNA (miRNA), long non-coding RNA (lncRNA), circular RNA (circRNA), small nuclear RNA, ribosomal RNA, and transfer RNA. In this review, we will mainly focus on the miRNA, lncRNA, and circRNA, which could mediate cellular processes including transcription, chromatin remodeling, post-transcriptional modifications, and signaling transduction 12. miRNA is a class of non-coding single-stranded RNAs with a length of 21-25 nucleotides. It regulates gene expression at the post-transcriptional level by forming the RNA-induced silencing complex (RISC), and resulting in either translational inhibition or mRNA degradation 13-15. lncRNA is a sort of RNA which contains more than 200 nucleotides in length, lacking of functional coding capacity. It has reported that lncRNA can regulate gene expression both in cis and in trans 16. circRNA is a sort of single-stranded closed circular RNA that lacks of 5′-3′ ends and poly(A) tails 17. The functions of circRNA mainly include sequestering miRNAs or proteins, regulating transcription, interfering splicing, and translating polypeptides 18.

Cancer stem cells are the important source of cancer stemness, they have the ability to self-renew, differentiate, and proliferate. Cancer stem cells should enable the tumor cells to self‑renew for generating new cells 19. And as we knew, the self-renewal ability of stem cells involved a rigidly choreographed flow, and may be a possible difficulty for the identification and eradication of cancer stem cells 20. Cancer stemness is likely to be one of the most crucial factors for cancer recurrence, metastasis, and drug resistance. Researches showed that the behaviors of cancer stem cells such as recurrence, relapse, metastasis, and resistance to therapeutic drugs are related to cancer progression 21, thus targeting cancer stemness is a very promising direction for cancer treatment.

Nowadays various therapeutic targets on cancer stemness have been explored. These targets include targeting the tumor microenvironment, cell surface markers, and signaling pathways. They are related to cancer stemness and cancer progression 22. A lot of cancer stem cells have well-established cell surface markers and the expression of stem cell specific surface markers have a connection with the increased resistance to treatment in cancers or be related to promoting cancer progression. For example, in hepatocellular carcinoma (HCC), CD13 was a marker of cancer stemness and was related to chemo-resistance and self-renewal 23. Similarly, CD44 promoted sorafenib resistance and tumorigenicity in HCC 24. It was showed that CD163 expressed highly in CD133-positive glioma stem cells, and contributed to gliomagenesis. Downregulation of CD163 expression decreased the expression of glioma stem-like cells (GSC) markers, such as CD133 and NANOG 25. CD9 was reported to have high migration potential and was positive associated with the resistance to chemotherapy drugs 26. In glioblastoma multiforme cells, CD133 was highly expressed and associated with tumorigenesis and drug resistance 27. In addition, epithelial cell adhesion molecule (EpCAM) was reported to promote colorectal cancer (CRC) metastasis and recurrence 28. Also, Nestin expression was related to the metastasis of breast cancer 29. Octamer-binding transcription factor 4 (OCT4), a key stemness transcription factor, was reported to be connected with tumorigenesis and resistance to chemotherapy 30, 31. These markers involved in cancer stemness, and may be potential therapeutic targets in many cancers.

So, in this review, we mainly focused on the functional roles of m6A modification and ncRNA in cancer stemness, illustrated the interaction between ncRNA and m6A modification in cancer stemness modulation through some key signaling pathways, and highlighted the clinical potential of ncRNA and m6A modifiers as promising biomarkers and therapeutic targets for indicating cancer stemness properties, so as to provide novel insights and avenues for finding therapeutic targets of cancer.

## The relationship of m6A modification and cancer stemness

**Figure 2:**
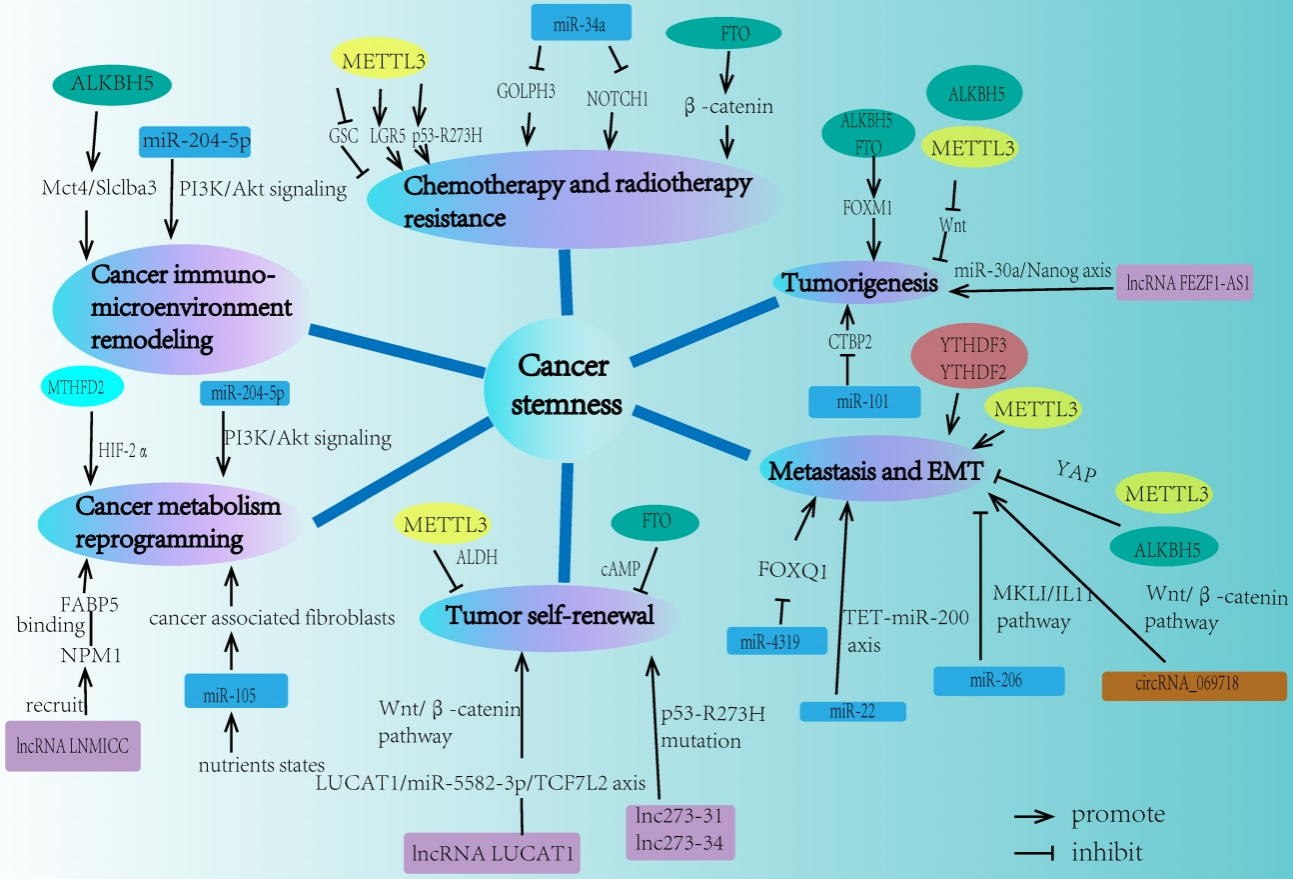
The network of m6A modification and ncRNA in regulating cancer stemness properties. Cancer stemness mainly contains chemo-resistance, radio-resistance, tumorigenesis, metastasis, tumor self-renewal, cancer metabolism reprogramming, and cancer immuno-microenvironment remodeling. m6A modification and ncRNA are both involved in each field of these cancer stemness aspects.

The properties of cancer stemness mainly consist of chemo-resistance, radio-resistance, tumorigenesis, metastasis, tumor self-renewal capacity, cancer metabolism reprogramming, and tumor immuno-microenvironment remodeling. Recent studies demonstrated that m6A modification played a significant role in different aspects of cancer stemness properties (**Fig. 2**).

### The resistance of chemotherapy and radiotherapy

It is well known that tumor burden, growth kinetics, heterogeneity, physical barriers, microenvironment, undruggable cancer drivers, and therapeutic pressures mainly determine drug resistance 32. Nowadays the relationship between m6A and chemo-resistance has been extensively studied 33. It was showed that m6A modification could enhance the resistance of drug treatment and lead to poor prognosis for cancers including osteosarcoma, colon cancer, and non-small-cell lung cancer (NSCLC). The studies also showed that the alteration of m6A methylation was closely related to cancer stemness, which could increase the acquired chemo-resistance in osteosarcoma cells 34. METTL3 was related to the acquisition of resistance to anticancer drugs and irradiation 35. Through upregulating LGR5, METTL3 participated in regulating stemness and chemosensitivity of colon cancer 36. METTL3 promoted a preferential pre-mRNA splicing and could promote drug resistance of colon cancer cells via increasing the expression of p53 R273H mutant protein 37. In another research, METTL3 was reported to enhance the translation of YAP mRNA by recruiting eIF3b and YTHDF1/3 to the translation initiation site. And downregulation of METTL3 expression could enhance cisplatin sensitivity in NSCLC cells 38. These findings suggested that m6A modifiers acted as oncogenes and m6A modification was an adverse factor for drug treatment in cancer, and increasing of m6A modification level enhanced the resistance of drug treatment. However, decreasing m6A modification level could also enhance the resistance of drug treatment. For example, FTO regulated the cervical squamous cell carcinoma and played an important role in enhancing the chemo-resistance through regulating the expression of β-catenin by reducing m6A modification levels in mRNA transcripts and increasing the activity of excision repair cross-complementation group 1 (ERCC1) 39. FTO upregulation and m6A hypomethylation increased TKI tolerance in leukemia treatment, through enhancing the mRNA stability of proliferation/survival transcripts containing m6A modification 40.

m6A modifiers could also acted as tumor suppressor genes in cancer development process. For instance, ALKBH5 could sensitize pancreatic cancer cells to gemcitabine through inactivating Wnt signaling by decreasing the level of m6A for Wnt inhibitory factor 1 (WIF-1) mRNA 41. In current studies, m6A modification showed its “double-edged sword” function in cancer based on the m6A modifiers and m6A level. m6A modifiers could both acted as oncogenes and suppressor genes, moreover, the upregulation and downregulation the level of m6A modification could both enhance the resistance of chemotherapy. However exact reasons are still not clear and worthy to conduct further research. Similarly, m6A modification also has effects on radio-resistance of cancers. For example, METTL3 silenced GSC so as to enhance sensitivity to γ-irradiation through SOX2-dependent DNA repair 42. The research about m6A and radio-resistance was far from enough, thus it might be a new research direction in the future for linking m6A modification and radio-resistance in cancer development.

### Tumorigenesis

Tumorigenesis is one of the most important traits of cancer stemness. Recent researches demonstrated that m6A modification could lead to tumor progression as well as tumor suppression. m6A modification suppressed tumorigenesis by altering mRNA behavior and changing the expression and activity of oncoprotein. For example, knockdown of METTL3 or METTL14, could not only dramatically promote GSC growth, self-renewal, and tumorigenesis, but also lead to a substantial increase of GSC-initiated tumor progression in transplanted mouse brains 43. ALKBH5 was reported to inhibit pancreatic cancer tumorigenesis by decreasing WIF-1 RNA methylation through mediating Wnt signaling 41.

However, on the other hand, m6A modification on target loci could also promote tumor formation 44. For instance, ALKBH5 maintained GSC tumorigenicity by sustaining FOXM1 expression 45. FTO mediating PKM2 demethylation so as to promote the tumorigenesis and development of liver cancer 41, 46.Another study demonstrated that FTO could promote melanoma tumorigenicity both in human and mice 47. EBV epi-transcriptome reprogramming by METTL14 was closely related to viral-associated tumorigenesis 48. m6A modification influenced tumorigenesis through m6A “writers” and “erasers”, and these m6A modifiers showed a “double-edged sword” function in cancer tumorigenesis. Actually, m6A readers also played an important role in tumorigenesis. For example, YTHDF1 promoted the translation of eIF3C by binding to m6A-modified eIF3C mRNA and increased the general translational output, so as to facilitating tumorigenesis of ovarian cancer 49. As showed above, the exact function of m6A modifier (“writer”, “eraser” and “reader”) in tumorigenesis was different from each other (including promoting tumorigenesis and inhibiting tumorigenesis). Further studies are needed to be carried out to find new strategies and treatments for preventing cancer progression.

### Metastasis and EMT

Metastasis makes the greatest contributions to the deaths of cancer patients. It is a kind of tumor cell movement and involves the adaptation and colonization of the cancer cells in the distant organ by invading the lymphatic vessels, blood vessels or body cavity 50. Epithelial-mesenchymal transition (EMT), a transition between epithelial and mesenchymal states, which allows cells to adopt the ability of migratory and invasive, has recently been identified as a crucial driver in tumor metastasis because of the significant similarity between cell plasticity in embryonic development and tumor progression 51.

As the most abundant modification in eukaryotes mRNA, m6A modifier was found to act as both oncogene and suppressor gene in cancer metastasis and the EMT process. And most researches indicated that m6A modifiers could act as oncogenes to promote cancer metastasis. For instance, YTHDF1 promoted the metastasis of HCC cell through the m6A-YTHDF1-EGFR axis 52. In osteosarcoma, low expression of FTO and METTL14 as well as high expression of METTL3 and YTHDF3 were associated with cancer metastasis and could be used as biomarkers to predict poor prognosis 53. Similarly, in liver cancer, YTHDF2 modulated the m6A methylation of OCT4 mRNA and promoted the metastasis of liver cancer stem cells 54. Furthermore, m6A modification in mRNA initiated by METTL3 directly promoted YAP translation and increased YAP activity by regulating the MALAT1-miR-1914-3p-YAP axis to induce NSCLC drug resistance and metastasis 55. METTL3-mediated m6A modification was critical for EMT and metastasis of gastric cancer (GC). Zinc finger MYM-type containing 1 (ZMYM1) was identified to be the m6A modification target of METTL3, and it facilitated the EMT programing and metastasis in GC through recruiting the CtBP/LSD1/CoREST complex to mediate the repression of E-cadherin promoter activity 56.

However, some studies indicated that m6A modifiers could also act as tumor suppressor gene to inhibit metastasis. In NSCLC, ALKBH5 inhibited tumor growth and metastasis by regulating the expression and activity of YAP 57. Additionally, METTL14-mediated m6A modification of SOX4 mRNA inhibited tumor metastasis in CRC 58. As showed above, m6A modifiers exert “double-edged sword” functions on metastasis, and the different function may be related to the different target genes or signaling pathways.

### Tumor self-renewal

The characteristic of self-renewal capacity is important for the driving force of tumorigenesis and metastasis. Recent researches indicated that m6A modification played an essential role in the self-renewal feature of cancer stemness.

The depletion of the METTL3 expression decreased ALDH activity and sphere-forming ability through a novel METTL3-AFF4-SOX2/MYC signaling axis, therefore inhibiting the self-renewal of bladder cancer stem cells 59. It was reported that knockdown of METTL3 or METTL14 could elevate CD44 expression and induce changes in m6A enrichment site of mRNA, which altered ADAM19 mRNA expression level with critical biological functions, so as to promote GSC growth and self-renewal 43. Additionally, FTO was verified to be a tumor suppressor and inhibited the stemness features of ovarian cancer. FTO could block cAMP signaling and inhibit ovarian cancer stem cell self-renewal. Depletion of FTO in ovarian cancer cells increased m6A modification, which induced ovarian cancer cell proliferation as spheres and promoted cancer stemness phenotype 60. Based on these findings, it was clear that m6A modification showed its “double-edged sword” function by regulating different signaling axis in tumor self-renewal. Thus, targeting m6A modified tumor self-renewal related signaling axis could be a novel avenue for cancer treatment.

### Cancer metabolism reprogramming

Through reprogramming nutrient acquisition and metabolic pathways, tumor could meet the bioenergy, biosynthesis, and redox requirements 61. The metabolism reprogramming activities are crucial characteristics of cancer development. It has been well known that cancer metabolism reprogramming involves glucose, fatty acid, and amino acid metabolism 62. There are no universal mechanisms to be used by all types of cancer cells to reprogram cancer metabolism. However, it seems to be several common mechanisms in glucose metabolism. For example, cancer cells can modulate and hijack several steps in glycolysis and glucose uptake to fulfill their anabolic demands 63. Some evidence showed that m6A modification may be closely related to metabolism reprogramming, which made it possible to find a novel insight to target on cancer metabolism.

The study indicated that MTHFD2 was involved in one-carbon metabolism, which linked m6A to the metabolic state of tumor cells in renal cell carcinoma patients, and led to a poor prognosis and induced late-stage disease. MTHFD2 regulated the m6A methylation levels of hypoxia-inducible factor-2 (HIF-2α) mRNA and enhanced HIF-2α-dependent metabolic reprogramming by regulating the epitopes' transcriptional landscape, which suggested that m6A methylation could promote aerobic glycolysis in a HIF-2α dependent manner 64. This study showed some connections about m6A modification and glucose metabolism. However, the researches of m6A modification in cancer metabolism reprogramming (such as fatty acid, and amino acid metabolism) are rarely studied, so there are a lot of work for us to excavate more details in this filed.

### Tumor immuno-microenvironment remodeling

Immune microenvironment remodeling could directly or indirectly affect the development of the tumor. The mechanisms mainly include promoting tumor angiogenesis, establishing an appropriate tumor microenvironment to promote tumor progression, regulating the functions of cancer stem cells, and selecting the tumor cells which are adapted to the microenvironment. At a large glance, tumor immune microenvironment (TIME) contains infiltrated-excluded, infiltrated-inflamed, and infiltrated-tertiary lymphoid structure, and each category has its own unique characteristics. Recent studies indicated that m6A modification has a close relationship with tumor immune-microenvironment remodeling. For example, in GC, the m6A modification plays a nonnegligible role in the formation of TIME diversity and complexity. Low m6A modification could increase mutation burden and immunity activation, which showed an inflamed tumor microenvironment phenotype and had a good survival rate. Whereas high m6A modification linked with a non-inflamed and immune-exclusion tumor microenvironment phenotype. This phenotype lacked of effective immune infiltration and exhibited a poorer survival rate for stroma 65. Moreover, research suggested that the deletion of m6A demethylase ALKBH5 could sensitize tumors to cancer immunotherapy. The mechanism was that ALKBH5 modulated Mct4/Slc16a3 expression, regulated lactate content of the tumor microenvironment, and adjusted the composition of tumor-infiltrating Treg cells and myeloid-derived suppressor cells 66. It was reported that m6A regulators such as ALKBH5 and YTHDF1 had a closely relation to the immune microenvironment remodeling of gliomas 67. Moreover, study revealed that the deletion of ALKBH5 decreased the infiltration of CD8+T cells, which linked m6A modification to the immune microenvironment of pancreatic cancer 68. Taken together, these findings uncovered a new direction of the function of RNA methylation in the tumor immuno-microenvironment and proved that m6A modification could be potential therapeutic targets in anticancer immunotherapy.

## The relationship of ncRNA and cancer stemness

With the development of ncRNAs research, accumulating evidence suggested that ncRNAs were closely related to cancer biological behaviors. Importantly, many ncRNAs played a crucial role in regulating cancer stemness (**Fig. 2**).

### miRNA

miRNAs dysregulation could break out the balance of cell signaling and growth processes, which provide a mechanism for the role of miRNAs in cancer process. Numerous studies have indicated that miRNA was associated with cancer stemness. In breast cancer, overexpression of miR-204-5p inhibited the proliferation and migration capacity of breast cancer cells. In addition, miR-204-5p overexpression altered the metabolic properties and suppressed the metastasis of breast cancer through regulating PI3K/Akt signaling pathway 69. In breast cancer, miR-105 was associated with metabolic reprogramming. When in a sufficient nutrients state, miR-105-reprogrammed cancer associated fibroblasts (CAFs), enhanced glucose and glutamine metabolism and offered energy to adjacent cancer cells. When nutrition was insufficient or there were much metabolic by-products accumulated, these CAFs could convert metabolic wastes, such as lactic acid and ammonium, into energy-rich metabolites 70. In breast cancer, miR-22 promoted aggressive metastatic capacity, and miR-22 could also silence antimetastatic miR-200, so as to inhibit the miR-200 promoter demethylation to exert the metastatic potential. In another word, miRNA-antagonism regulated breast cancer stemness through chromatin remodeling 71. Moreover, targeting miR-34a-NOTCH1 axis could reduce breast cancer stemness and chemo-resistance 72. miR-206/TWF1/MKL1-SRF/IL-11 signaling pathway played an important role in breast cancer initiation and progression. Targeting MKL1/IL-11 pathway, miR-206 could suppress breast cancer stemness and metastasis 73.

Furthermore, in bladder cancer, downregulation of miR-34a resulted in GOLPH3 overexpression, and ectopic expression of miR-34a decreased the stem cell properties of chemo-resistant urothelial bladder cancer cells. Thus, targeting miR-34a-GOLPH3 axis could reduce bladder cancer stemness especially in drug resistance context 74. In colon cancer, miR-3120-5p was highly expressed in the population of CD133+ and LGR5+ stem cells. Through targeting Axin2, miR-3120-5p promoted stemness and invasiveness of colon cancer 75. In HCC, a huge number of studies have convinced that dysregulated miRNAs took part in the carcinogenesis and aggressiveness of HCC. For instance, miR-4319 repressed cell proliferation, accelerated apoptosis, inhibited EMT and prevented cancer stemness by targeting FOXQ1 76. In ovarian carcinoma, myeloid-derived suppressor cells triggered the expression of miR-101, so as to repressing the expression of co-repressor gene C-terminal binding protein-2 (CtBP2). Consequently, miR-101 was related to increasing metastatic capacities and promoting tumorigenesis and cancer stemness 77. By shedding light on cancer stemness-related miRNA at the molecular level, it might provide a novel avenue for targeting cancer stemness (including cell proliferation and migration, altering metabolic state, chemo-resistance, and metastasis in breast cancer, bladder cancer and HCC) and improving the efficacy of cancer treatment.

### lncRNA

The dysregulated expression of lncRNA in cancer could regulate cancer stemness such as self-renewal, cancer metabolism reprogramming, and tumor progression. For example, in NSCLC, lncRNA played an important role in cell cycle, proliferation, immune responses, and metastasis. Knockdown of LINC-ITGB1 could suppress cancer stem cell sphere formation and inhibit the expressions of stemness-associated genes, so as to reduce the invasive and migratory potential of NSCLC 78. In CRC, the p53-R273H mutation could enhance cancer stemness and chemo-resistance through regulating specific lncRNAs such as lnc273-31 and lnc273-34 79. Also, lncRNA LNMICC could recruit the nuclear factor NPM1 to fatty acid binding protein (FABP5), so as to reprograming fatty acid metabolism and further promoting metastasis 80.

Current evidence revealed that lncRNA regulated cancer stemness via forming different axis or signaling pathways. For example, LINC-PINT regulated laryngeal carcinoma stemness through miR-425-5p/PTCH1/SHH axis 81. In breast cancer, through targeting the miR-30a/Nanog axis**,** lncRNA FEZF1-AS1 promoted breast cancer stemness and tumorigenesis 82. In addition, LINC00511 could promote proliferation, sphere-formation ability, and elevate some stemness markers expression such as OCT4, Nanog, and SOX2 in breast cancer. Besides, LINC00511 promoted tumor growth by inducing the miR-185-3p/E2F1/Nanog axis 83. lncRNA small nucleolar RNA host gene 20 (SNHG20) was verified to be an oncogene in several cancers. Knockdown of SNHG20 could suppress cell proliferation, increase cell apoptosis, and impair stemness properties, therefore preventing cancer progression. In detail, through activating PI3K/AKT/mTOR signaling pathway, lncRNA SNHG20 promoted tumorigenesis and cancer stemness in glioblastoma 84. Moreover, it was demonstrated that upregulation of lncRNA LUCAT1 promoted breast cancer stem cell proliferation, while LUCAT1 downregulation inhibited the self-renewal of breast cancer stem cells. Further studies revealed that LUCAT1 increased stem-like properties of breast cancer stem cell, and lncRNA LUCAT1/miR-5582-3p/TCF7L2 axis modulated breast cancer stemness via Wnt/β-catenin pathway 85. So, focusing on the essential signaling pathways might provide novel insights for cancer stemness therapy.

### circRNA

circRNA has great pre-clinical diagnostic and therapeutic potentials in cancers. It was reported that circRNA was closely related to cancer stemness in the aspects of proliferation, migration, metastasis, and chemo-resistance. The well-known regulatory mechanism of circRNA in cancers was acting as a miRNA sponge and competing for mRNA-binding sites on miRNAs. For example, in breast cancer, circRNA_100876 promoted cancer metastasis and increased cancer proliferative capacity by modulating microRNA-361-3p expression in a miRNA sponge way 86. In addition, hsa_circ_0025202 acted as a miRNA sponge for miR-182-5p and suppressed breast cancer growth by regulating the expression and activity of FOXO3a 87. In cervical cancer, hsa_circRNA_101996 served as a sponge of miR-8075, which targeted TPX2 and inhibited cancer cell proliferation, migration, and invasion 88. circSLC8A1 acted as a miRNA sponge of miR-130b/miR-494 and regulated the expression of their target gene PTEN, so as to suppressing the progression of bladder cancer 89. Besides acting as a miRNA sponge, recent researches revealed novel mechanisms of circRNA in cancer stemness regulation. For instance, in HCC, circZKSCAN1 was reported to suppress cancer stemness through regulating the function of the RBP fragile X mental retardation protein (FMRP). And the downstream target gene of FMRP was cell cycle and apoptosis regulator 1 (CCAR1). CCAR1 could enhance cancer stemness through Wnt/β-catenin signaling pathway 90. However, inhibition of circRNA_069718 could reduce the expression levels of Wnt/β-catenin pathway-related genes such as β-catenin, c-myc, and cyclin D1, consequently reducing triple-negative breast cancer cells proliferation and invasion ability 91. Furthermore, the Hippo-YAP signaling pathway was identified to be activated by circPPP1R12A-73aa. And this signaling pathway could promote the growth and metastasis of colon cancer 92. Overall, these evidences indicated that circRNAs played an essential role in cancer stemness modulation. However, the complexity structure and function of circRNA make it difficult to fully understand its role in cancer stemness. Therefore, more extensive mechanism researches of circRNA are needed to conduct in the future.

## The interplay between ncRNA and m6A modification

**Figure 3:**
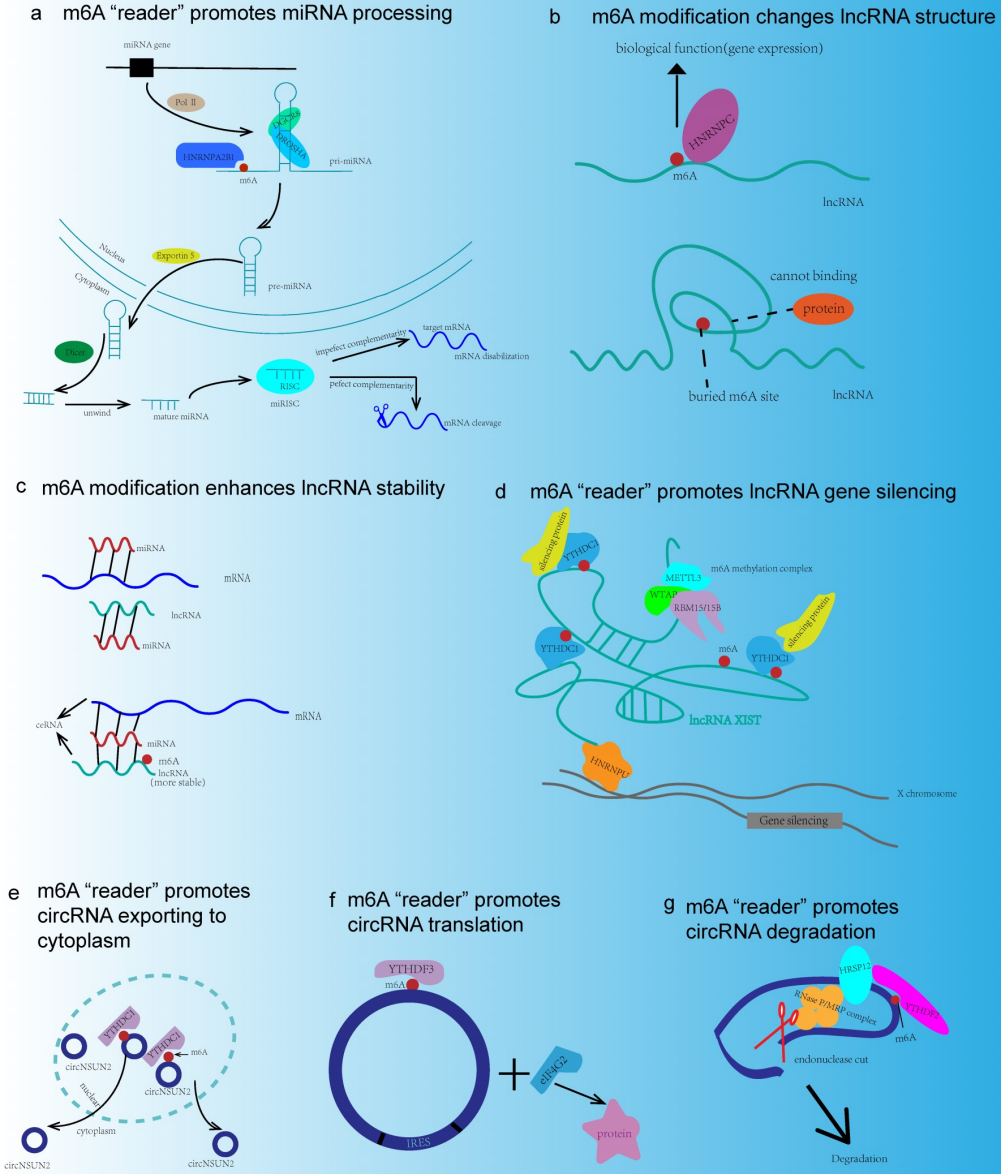
The interactions between m6A modification and ncRNA. (a) m6A “reader” HNRNPA2B1 binds to pri-miRNA and interacts with DROSHA/DGCR8 complex or increases Dicer splicing, so as to promote miRNA processing. (b) m6A modification changes lncRNA structure in order to expose the m6A binding site and recruit RNA binding proteins to exert their biological functions. (c) lncRNA acts as ceRNA and m6A modification increases the stability of lncRNA. (d) HNRNPU anchors XIST to the X chromosome. XIST recruits m6A methylation complex and results in m6A methylation around. m6A “reader” YTHDC1 binds to XIST and promotes gene silencing. (e) YTHDC1 binds to circNSUN2 and promotes circNSUN2 exporting to cytoplasm. (f) circRNA translation is a cap-independent way involving YTHDF3, eIF4G2, and IRES. (g) m6A site could be recognized by YTHDF2, and m6A recruits HRSP12 to promote circRNA degradation through YTHDF2-HRSP12-RNase P/MRP axis.

As the most abundant mRNA modification in mammals, m6A modification was closely related to the functions of ncRNA, and the interplay mechanisms between ncRNA and m6A modification were illustrated as follows (**Fig. 3**).

### miRNA and m6A modification

The interplay between miRNA and m6A modification has recently been observed. On the one hand, m6A modification participated in cancer progression regulation by affecting miRNA biogenesis or stability. Mechanism research demonstrated that m6A modification promoted miRNA biogenesis mainly through the interactions between m6A and DGCR8 93 (**Fig. 3a**). For example, in bladder cancer, METTL3 accelerated the maturation of pri-miR221/222 in an m6A-dependent manner 94. m6A modification in pri-miRNA allowed DGCR8 to bind specific substrates and further promoted miRNA maturation. Moreover, the maturation of miRNA resulted in the reduction of PTEN, which led to cancer cell proliferation and tumor growth 93. Additionally, in HCC, METTL14 could also regulate the maturation of miRNA. Mechanistically, METTL14 promoted pri-miR-126 processing by recognizing DGCR8 and binding DGCR8 to pri-miRNAs, and thereby suppressing the metastasis of HCC 95. It was reported that the RNA-binding protein HNRNPA2B1 also had a strong interaction with pri-miRNA. In NSCLC, HNRNPA2B1 bound to m6A modification site in pri-miRNA transcripts, so as to interact with the miRNA microprocessor complex protein DGCR8, thus promoting pri-miR-106b processing 96. In glioblastoma, HNRNPC, an m6A “reader”, bound to pri-miR-21 to promote the maturation of miR-21. Silencing of HNRNPC reduced miR-21 expression and suppressed the AKT-p70S6K pathway and inhibited cancer invasion 97. Another mechanism about m6A modification regulating miRNA biogenesis was to increasing the splicing of Dicer for pre-miRNA. For example, in NSCLC, METTL3 regulated the Dicer cleavage of pre-miR-143-3p through the miR-143-3p/VASH1 axis and played an essential role in cancer metastasis 98. Additionally, m6A “eraser” also involved in miRNA processing. There were some positive correlations between m6A “eraser” and miRNA expression level. For instance, knockdown of FTO could upregulate the m6A modification level, and further resulted in the downregulation of some mature miRNAs such as miR-7-5p and miR-22-3p 99. The detailed mechanism of this regulation was worthwhile to do further studies (**Table 1**).

On the other hand, miRNA could modulate m6A abundance on their target transcripts. For instance, miRNA could regulate m6A modification levels through modulating the binding of METTL3 and mRNAs which containing miRNA targeting sites 100. Moreover, miRNA could regulate the expression levels of m6A modifiers so as to significantly influence the whole process of m6A modification. For example, miR-33a inhibited the expression of METTL3 by targeting its mRNA and suppressed cell proliferation in NSCLC 101. In addition, miR-600 was reported to inhibit the expression of METTL3 and prevent NSCLC progression by reversing the biological functions of METTL3 102. In breast cancer, hepatitis B X-interacting protein (HBXIP) upregulated METTL3 by suppressing let-7g. Moreover, METTL3 could increase the expression of HBXIP, thus forming a positive feedback loop of HBXIP/let-7g/METTL3/HBXIP to accelerate cancer cell proliferation 103. Additionally, miR-145 inhibited YTHDF2 expression by targeting the 3'-UTR of YTHDF2 mRNA and suppressed cell proliferation in HCC 104. In CRC, low expression level of miRNA-1266 promoted cancer occurrence and progression by directly targeting FTO, therefore linking m6A modification and miRNA regulation in cancer development together 105 (**Table 2**).

In conclusion, the interplay between m6A modification and miRNA has been noticed. On the one hand, m6A modification influenced the biogenesis and stability of miRNA through (1) the interactions between m6A and DGCR8, (2) the interactions between m6A and RNA-binding protein HNRNPA2B1, (3) increasing the splicing of Dicer for pre-miRNA. On the other hand, miRNA could modulate m6A abundance on their target transcripts and miRNA could regulate the expression levels of m6A modifiers through targeting at their 3'-UTR.

### lncRNA and m6A modification

It was reported that m6A modification was also common in lncRNA. And the interactions between lncRNA and m6A modification have been extensively investigated. For example, the m6A switch played an important role in this interaction (**Fig. 3b**). m6A modification affected lncRNA-protein interactions in the biological process by altering RNA structure. m6A could change the local structure in mRNA and lncRNA so as to facilitate their binding to HNRNPC, and consequently, m6A modification was responsible for pre-mRNA processing. Besides, METTL3 and METTL14 enhanced the binding of lncRNA MALAT1 and HNRNPC to promote gene expression 106. Moreover, m6A participated in the lncRNA-mediated ceRNA model (**Fig. 3c**). Specially, the study showed that METTL3/YTHDF3 complex could increase the stability of MALAT1, and promote the translation of YAP mRNA through the MALAT1-miR-1914-3p axis. MALAT1 functioned as a ceRNA and sponged miR-1914-3p to promote the invasion and metastasis of NSCLC 107. Furthermore, m6A modification was involved in the lncRNA-miRNA interactions. It was reported that m6A modification of LINC1281 mediated a ceRNA model to regulate mouse embryonic stem cells differentiation. Interestingly, down-regulation of METTL3 could abolish the binding of let-7 to LINC1281, thus this RNA-RNA interaction was associated with m6A modification 108. However, the underlying mechanism of this interaction needed further investigation. Additionally, knockdown of METTL3 could impair XIST-mediated gene silencing, which was another regulatory model of m6A modification in lncRNA 109 (**Fig. 3d**) (**Table 1**).

Meanwhile, lncRNA could also regulate the expressions and functions of m6A modification molecules. In cervical cancer, lncRNA GAS5-AS1 was proved to increase the stability of GAS5 by promoting the ALKBH5-dependent m6A demethylation 110. In liver cancer, the lncRNA GATA3-AS, acted as a guide to facilitate KIAA1429-mediated m6A modification, and thereby promoting the cancer growth and metastasis 111. Furthermore, in GC, lncRNA LINC00470 promoted METTL3-mediated m6A methylation so as to inhibiting the expression of PTEN, which contributed to the development of GC 112 (**Table 2**).

The interaction between m6A modification and lncRNA involved two major aspects. m6A modification affected RNA-protein interactions, participated in the lncRNA-mediated ceRNA model, and involved in the lncRNA-miRNA interactions. Moreover, lncRNA could also regulate the expressions and functions of m6A modification by modulating the m6A modifiers.

### circRNA and m6A modification

m6A modification was also a popular phenomenon in circRNA. Despite sharing the same “readers” and “writers” of m6A modification in both circRNAs and mRNAs, the m6A modification of circRNAs often derived from exons which have no methylation in mRNAs, whereas mRNAs that are methylated on the same exons that compose m6A modification, circRNAs become less stable when recognized by reader YTHDF2 113. Accumulating evidence suggested that m6A modification could impact lots of biological functions of circRNA, such as promoting cytoplasmic export of circRNA, driving translation, and mediating degradation. For example, m6A modification of circNSUN2 modulated its cytoplasmic export (**Fig. 3e**). The mechanism was that YTHDC1 recognized this circNSUN2 and then formed a circNSUN2/IGF2BP2/HMGA2 RNA-protein ternary complex in the cytoplasm. In addition, this m6A modified circNSUN2 could enhance the stability of HMGA2 mRNA, which promoted CRC metastasis and progression 114. Moreover, m6A modification could promote the cap-independent protein translation of some circRNAs through internal ribosomal entry sites (IRESs). This kind of m6A-driven translation required initiation factor eIF4G2 and m6A “reader” YTHDF3, which largely increased the knowledge of the human transcriptome 115 (**Fig. 3f**). Recently, a subset of circRNAs modified by m6A could be recognized by YTHDF2 and the levels of circRNAs were selectively downregulated via the HRSP12-RNase P/MRP endoribonuclease complex, which might serve as a new mechanism for m6A modification mediated degradation of circRNAs 116 (**Fig. 3g**) (**Table 1**).

As showed above, m6A modification could impact the functions of circRNA. These functions include promoting cytoplasmic export, driving translation, and mediating degradation. However, the influence about circRNA on m6A modification was not reported up to date, which might be a novel research direction in the future.

## The relationship of ncRNA modified by m6A and cancer stemness

As indicated above, we summarized the relationship among m6A modification, ncRNA, and cancer stemness. However, whether there were any connections between m6A modified ncRNA and cancer stemness remained unclear. So in this part, we illustrated the relationship of ncRNA modified by m6A and cancer stemness.

m6A modification in miRNA played a crucial role in cancer stemness. In bladder cancer, METTL3 promoted the maturation of miR-221/222 and accelerated cancer cell proliferation, growth, and stemness 94. In CRC, METTL3 promoted the maturation of miR-1246 and downregulated suppressor gene SPRED2. SPRED2 was the downstream target of miR-1246, and downregulation of SPRED2 could reverse the inhibition of MAPK pathway, thus promoting cancer metastasis and stemness 117. There were also some evidences indicated that m6A modification in lncRNA and circRNA exhibited a strong connection with cancer stemness. For example, in nasopharyngeal carcinoma, lncRNA FAM225A could sponge miR-590-3p/miR-1275 to exert its biological functions. Through activating the FAK/PI3K/AKT signaling pathway, m6A modification made the oncogenic lncRNA FAM225A more stable, therefore promoting tumorigenesis, metastasis and stemness 118. In CRC, m6A modification accelerated the circNSUN2 exporting to the cytoplasm, and the upregulation of circNSUN2 in the cytoplasm enhanced cancer invasion and stemness. The detailed mechanism was that the circNSUN2/IGF2BP2/HMGA2 complex could stabilize HMGA2, and HMGA2 mRNA was closely related to the invasion of CRC cells 114. In HCC, m6A modified circRNA-SORE sustained sorafenib resistance by regulating Wnt/β-catenin signaling. circRNA-SORE acted as a miRNA sponge, and sequestered miR-103a-2-5p and miR-660-3p, thereby competitively activating the Wnt/β-catenin pathway to inducing sorafenib resistance and stemness 119. To sum up, it was obvious that m6A modified ncRNA played an essential role in the modulation of cancer stemness, and the underlying mechanisms and clinical significances of this regulation were worthwhile to conduct further investigations.

## Key signaling pathways regulated by ncRNA and m6A modification in cancer stemness

It was confirmed that METTL3 could methylate pri-miR-1246 to promote its maturation. And the METTL3/miR-1246/SPRED2 axis played a significant role in CRC metastasis through regulating MAPK signaling pathway 117. METTL14 inhibited CRC cell growth, migration and invasion via miR-375/Yes-associated protein 1 (YAP1) pathway and miR-375/SP1 pathway 120. miR-186 was a direct target of METTL3, and via Wnt/β-catenin signaling pathway, METTL3/miR-186 could promote the progression of hepatoblastoma 121. As showed above, there were some evidences that ncRNAs modified by m6A played a significant role in cancer stemness modulation, however, the number of these researches were limited. To understand the key signaling pathways regulated by ncRNA and m6A modification in cancer stemness, some key molecules in these pathways might provide novel insights and avenues.

**Figure 4:**
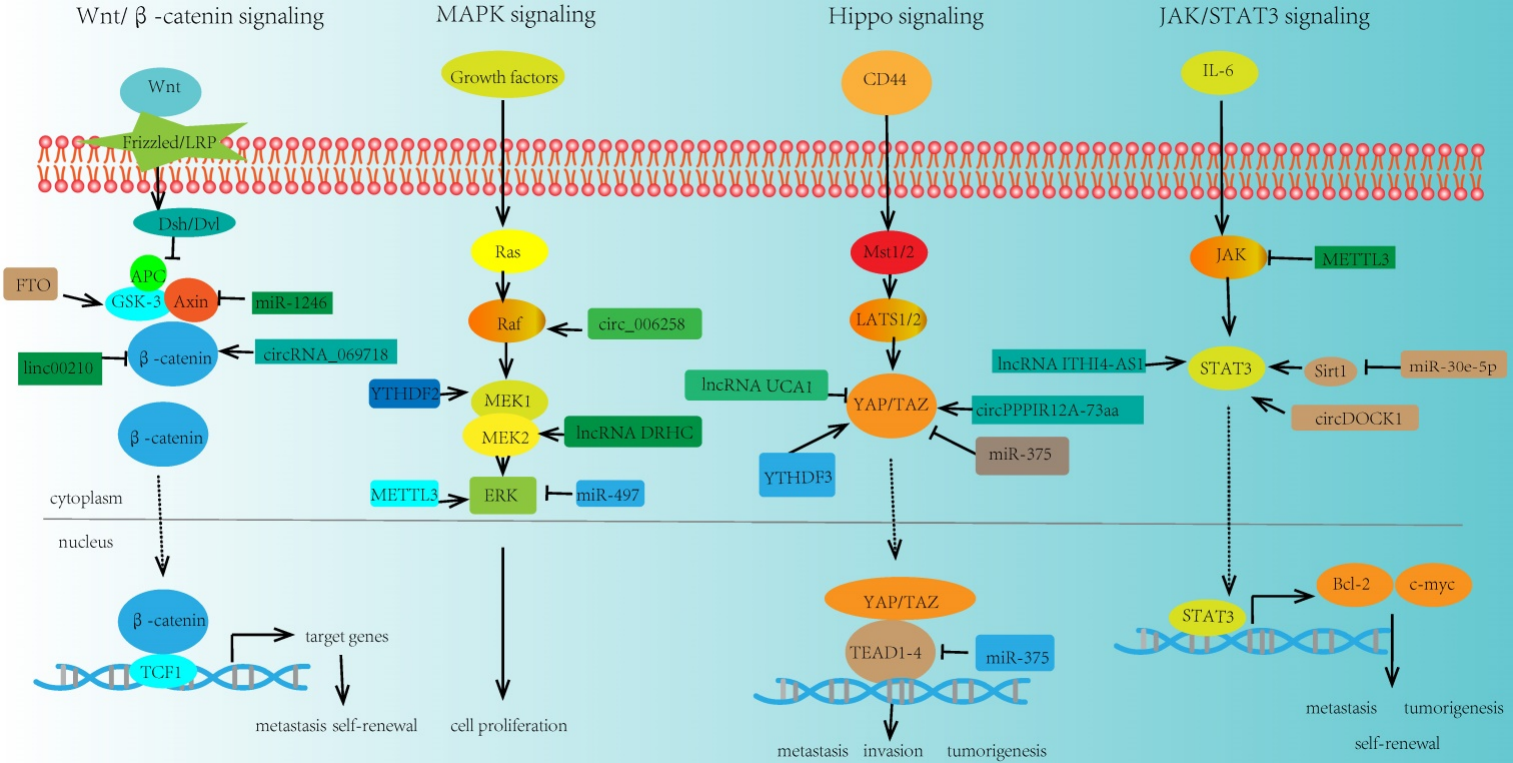
Key signaling pathways regulated by ncRNA and m6A modifiers in cancer stemness. m6A modification can modulate some key molecules in cancer stemness related signaling pathways, and the expressions and functions of these molecules could also be regulated by ncRNA, thus linking m6A modification, ncRNA, and cancer stemness together.

Numerous researches demonstrated that some key signaling pathways were involved in the regulation of cancer stemness through the interactions between ncRNA and m6A modification on the essential molecules of these pathways (**Fig. 4**).

### Wnt/β-catenin signaling

Wnt signaling was a key driver of cancer stemness properties. The core Wnt/β-catenin signaling pathway, could control stem cells, and contribute to cancers, such as CRC, HCC, and pancreas cancer 122. In addition, ncRNAs were verified to play a significant role in this signaling pathway. For example, miR-1246 could activate Wnt/β-catenin pathway by suppressing the expression of AXIN2 and glycogen synthase kinase 3β (GSK3β) 123. lncRNA NEAT1 activated Wnt/β-catenin signaling pathway via DDX5 and fulfilled its oncogenic functions to promote CRC progression and metastasis 124. LINC00210 activated Wnt/β-catenin signaling through interacting with CTNNBIP1 and blocking the combination between CTNNBIP1 and β-catenin, so as to promoting the self-renewal and propagation of liver cancer 125. Inhibition of circRNA_069718 could reduce the expression level of β-catenin in the Wnt/β-catenin pathway 91. Moreover, some key molecules in this signaling pathway such as GSK3β was associated with m6A modification. For instance, FTO was elevated in GSK3β-deficient mESCs. FTO could be phosphorylated by GSK3β in Wnt/β-catenin pathway. However, in GSK3β knockout mESCs, that process is impaired, which leads to the upregulation of FTO protein. And this result may provide a novel insight in the interplay between m6A modification and signaling pathway 126.

### MAPK signaling

MAPK signaling pathway was also closely related to cancer stemness. And various ncRNAs participated in the modulation of this signaling pathway. Upregulation of miRNA-21 expression could suppress the MAPK signaling in CVB3-infected Hela cells 127. miR-497 inhibited the expression of the MAPK/ERK signaling pathway and promoted the apoptosis of osteosarcoma cells 128. lncRNA DRHC was reported to inhibit the tumorigenicity of HCC through modulating MEK/ERK activities in MAPK signaling 129, 130. miR-125a-3p impaired p38/MAPK pathway and exhibited tumor-suppressive functions. In addition, circ-MAPK4 acted as a sponge of miR-125a-3p and affected glioma proliferation and apoptosis by regulating p38/MAPK pathway 131. In breast cancer, circ_0006528 could promote cancer growth and migration by promoting the expression of Raf1 in MAPK/ERK pathway 132. MEK1/2 and ERK1/2 were two essential molecules in MAPK signaling pathway. It was reported that m6A modification had crucial effects on these molecules. For example, overexpression of YTHDF2 activated MEK and ERK in HCC cells and suppressed cancer cell proliferation. The mechanism was that YTHDF2 acted as a tumor suppressor and bound the m6A modification site of EGFR 3'-UTR, thus promoting the EGFR mRNA degradation 133. Moreover, METTL3 depletion could increase the phosphorylation of ERK in MAPK signaling pathway and influence the whole activity and function of this pathway 134.

### Hippo signaling

The role of Hippo signaling in cancer stemness has already been observed. In GC, miR-375 was revealed to regulate Hippo pathway through targeting the YAP1-TEAD4-CTGF axis. Further study suggested that overexpression of YAP1 could reduce the tumor suppressive effect of miR-375, and promote GC progression 135. Besides, lncRNA UCA1 was reported to promote pancreatic cancer cell migration and invasion through Hippo pathway. Overexpression of UCA1 could inhibit the phosphorylation of YAP in the Hippo pathway and increase the YAP expression level 136. Similarly, in GC cells, LINC00662 regulated GC cell proliferation via Hippo pathway. Knockdown of LINC00662 could suppress Hippo-YAP1 signaling pathway through sponging miR-497-5p 137. Another study indicated that circPPP1R12A-73aa could activate Hippo-YAP signaling pathway so as to promote colon cancer metastasis 92. Meanwhile, m6A modification was also involved in the modulation of key molecule YAP in the Hippo signaling. YTHDF3 was a novel target of YAP, which could facilitate the degradation of m6A modified lncRNA GAS5, thus uncovering a negative functional loop of lncRNA GAS5-YAP-YTHDF3 axis, and identifying a novel mechanism for m6A induced decay of GAS5 on YAP signaling in the progression of colorectal cancer 138.

### JAK/STAT3 signaling

JAK/STAT3 signaling pathway is one of the most promising pathways in cancer stemness research field, which bridges a linkage between ncRNA and m6A in tumorigenesis and metastasis. In NSCLC, miR-30e-5p suppressed tumorigenesis and metastasis by inhibiting USP22-mediated Sirt1/JAK/STAT3 signaling. Further study demonstrated that miR-30e-5p could inhibit Sirt1 expression and increase the expression of phosphorylated STAT3, so as to activate STAT3 activity 139. In addition, in CRC, the upregulation of lncRNA ITIH4-AS1 induced the downregulation or depletion of RE1 silencing transcription factor (REST). The downregulation of REST further enhanced ITIH4-AS1 expression and promoted tumor proliferation and metastasis through JAK/STAT3 pathway 140. Moreover, circDOCK1 was reported to promote the phosphorylation of JAK1 and STAT3 in JAK/STAT3 signaling pathway and accelerate thyroid carcinogenesis through the inhibition of miR-124 141. Recently, there were some evidences about m6A modification in regulating the key molecules in JAK/STAT3 signaling pathway. For instance, loss of METTL3 interfered the expression of JAK2 and SOCS3, impaired self-renewal capacity, and triggered the differentiation of induced pluripotent stem cells 142.

To sum up, cancer stemness could be regulated by some key signaling pathways, and the essential molecules in these signaling pathways could be associated with m6A modification and ncRNA modulation. The above evidence provided a novel bridge between the m6A modification and ncRNA in cancer stemness, especially in cancer invasion, metastasis, tumorigenesis, and self-renewal. That is to say, m6A modification regulated or interacted with some key molecules in cancer stemness related signaling pathways, and these pathways could also be influenced by ncRNA, which linked m6A modification, ncRNA, and cancer stemness together. However, the interactions of m6A modification with the key molecules in signaling pathways have not been well studied, and the exact mechanisms of these regulations still required further investigation.

## The clinical potential of ncRNAs and m6A modifiers as promising biomarkers for indicating cancer stemness properties

**Figure 5:**
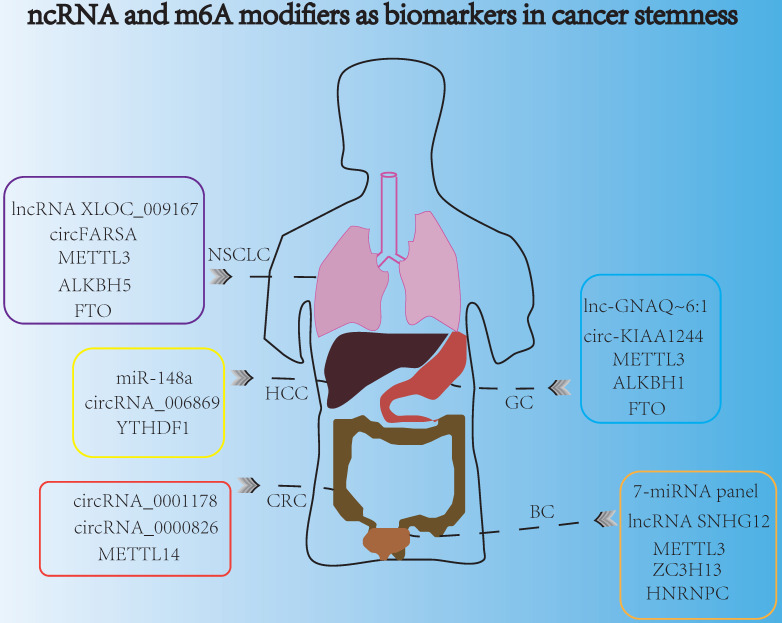
ncRNA and m6A modifiers are promising biomarkers for indicating cancer stemness.

Late detection was the main reason leading to the poor survival and prognosis of cancer patients. Highly specific and sensitive biomarkers might be a new direction for developing non-invasive cancer diagnosis markers for early detection. Nowadays, ncRNAs and m6A modifiers could act as promising biomarkers for indicating cancer stemness and cancer progression (**Fig. 5**). For example, exosomal miRNAs served as new biomarkers for early and minimally invasive cancer diagnosis, because exosomes were usually stable and could exist in body fluids, such as blood, saliva, urine, latex, and cerebrospinal fluid 143. Recently, circulating miRNA profiles were used as promising tools for liquid biopsy in cancer screening, and the 7-miRNA panel (7-miRNA panel: miR-6087, miR-6724-5p, miR-3960, miR-1343-5p, miR-1185-1-3p, miR-6831-5p and miR-4695-5p) were reported to act as non-invasive biomarkers for the early detection of bladder cancer 144. Moreover, detecting the miRNA biomarkers in plasma had a potential diagnostic value for the screening of HCC. Research indicated that peripheral plasma miR-148a was convinced to be a promising biomarker to predict HCC occurrence and progression 145. In addition, aberrant levels of lncRNAs could also determine the invasive and metastatic capacities of cancer cells, and lncRNA SNHG12 was a promising biomarker with clinical potential in many cancers including GC, NSCLC, and bladder cancer, because of its high stability and abundantly expression in multiple tumors 146. Some exosome-derived lncRNAs were involved in the modulation of cancer stemness aspects such as chemotherapeutic drug resistance 147. Additionally, exosomal lncRNAs derived from tumors were suitable candidates for non-invasive diagnosis. For instance, serum exosomal lnc-GNAQ-6:1 might be evaluated as a novel diagnostic marker for gastric cancer 148. More interestingly, circulating lncRNA XLOC_009167 was stable in whole blood under different conditions, so lncRNA XLOC_009167 may have a better diagnostic potential than traditional biomarkers in the diagnose of NSCLC 149. In addition, the expression of circRNA_0001178 and circRNA_0000826 were different in CRC patients with and without liver metastasis, which indicated that these two circRNAs might act as different diagnostic biomarkers for liver metastasis in CRC 150. Moreover, circ-KIAA1244 was reported to be detected in GC patients' plasma exosomes, and low expression of plasma circ-KIAA1244 could inhibit lymphatic metastasis and increase overall survival rate, which indicated that circ-KIAA1244 might serve as a novel circulating biomarker for the detection of GC metastasis 151. Furthermore, the expression of circ_0068669 was associated with the microvascular invasion and TNM stages, suggesting that it could act as a promising biomarker for the prediction of HCC metastasis 152. In NSCLC, some evidence indicated that the plasma circFARSA could serve as a potential non-invasive biomarker for cancer invasion and metastasis 153.

Similarly, m6A modifiers were also reported to serve as biomarkers in cancer stemness, due to their significant regulatory roles in modulating gene expressions, during cancer initiation, metastasis and recurrence. Specifically, downregulation of METTL14 expression could facilitate tumor metastasis, and METTL14 was verified to be a potential prognostic biomarker for indicating the metastasis of CRC. Moreover, METTL14 also acted as an effective therapeutic target of CRC, which influenced the SOX4-mediated EMT process and PI3K/AKT signaling to inhibit CRC progression 58. Besides, METTL3 was reported to be associated with poor prognosis and survival. Overexpression of METTL3 could promote cell proliferation, angiogenesis, glycolysis process and liver metastasis in GC 154. Furthermore, the aberrant expressions of m6A “erasers” such as FTO and ALKBH1 both had distinct prognostic values in GC patients, and these two “erasers” might be used as potential biomarkers for indicating GC progression and metastasis 155. In addition, m6A “readers” such as YTHDF1 and HNRNPC also served as promising biomarkers in cancer stemness aspects. In HCC, YTHDF1 was reported to play an important role in regulating cell cycle progression and cancer metabolism reprogramming, suggesting that YTHDF1 might be a potential biomarker for the prognosis of HCC 156. Additionally, HNRNPC participated in the malignant behavior such as metastasis and invasion of bladder cancer, and the expression of HNRNPC was dramatically associated with different clinicopathological variables of bladder cancer patients, which indicated it may be promising biomarker 157. Recently, peripheral blood m6A modifier seems to be a potential noninvasive biomarker for the detection and diagnosis of NSCLC, and down-regulating FTO and ALKBH5 in NSCLC had a great connection with tumor stage and metastasis 158.

Nowadays, some m6A inhibitors are explored, which provide novel treatments for cancers, through targeting m6A regulators. Most inhibitors targeted at m6A “erasers”. For example, MO-I-500 was a FTO inhibitor, which inhibited the survival and colony formation of breast cancer cell 159. R-2HG, which was known as a metabolic product of mutant IDH1/2, was defined as an inhibitor of FTO and inhibited leukemia progression 160. FB23-2 (an inhibitor of FTO) suppressed proliferation and promoted the apoptosis of AML cell 161. FTO-04 increased m6A modification levels in glioblastoma stem cells through inhibiting of FTO 162. In addition, there are other FTO inhibitors, such as Meclofenamic acid (MA) was applied to cancer treatments 163. Moreover, ALKBH5 inhibitors were also been developed. For example, MV1035 was an inhibitor of ALKBH5, and it significantly reduced the migration and invasiveness of glioblastoma 164. m6A inhibitors showed important functions in various cancers, as they could took part in the development of cancers through inhibiting m6A modifiers. However, the inhibitors of m6A “writers” and “readers” have not been reported up to date, which will be a novel research direction in the future.

## Conclusions and limitations

In summary, m6A modification and ncRNA (miRNA, lncRNA, circRNA) are closely related to cancer stemness properties (chemo-resistance, radio-resistance, tumorigenesis, metastasis, tumor self-renewal capacity, cancer metabolism reprogramming, and tumor immuno-microenvironment remodeling). And the interaction among m6A modification, ncRNA and cancer stemness were quite complex. The m6A modifiers could both act as oncogene and tumor suppressor gene, ncRNAs could exert different functions among multiple cancers. Besides, the increasing and decreasing of m6A modification level could both be cancer-promoting factors, and the exact mechanisms still not clear and may be associated with different targets or signaling pathways. We illustrated the interplay between ncRNA and m6A modification in cancer stemness modulation and demonstrated that some key signaling pathways might act as bridges in this interplay. In addition, m6A modifiers and ncRNAs might serve as promising biomarkers for indicating cancer stemness properties, as they can offer an early and non-invasive way for cancer detection and provide novel insights for finding useful targets for cancer diagnosis, treatment, and prognosis. Furthermore, some m6A inhibitors were explored and may be novel therapeutic treatments for cancer stemness. More importantly, although some researches indicated that ncRNAs could be potential biomarkers for cancer diagnosis, very few of them have completed clinical trials, which means that there are still a long way to go to verify their clinical values.

## Figures and Tables

**Table 1:** m6A modification in ncRNAs

m6A modifiers	ncRNAs	Mechanisms	Notes	Cancer types/functions	References
METTL3	miR-221/222	Promote miRNA biogenesis	m6A allow DGCR8 to bind specific substates	Bladder cancer	94
METTL14	miR-126	Promote miRNA biogenesis	m6A allow DGCR8 to bind specific substates	HCC	95
HNRNPA2B	miR-106b	Promote miRNA biogenesis	m6A allow DGCR8 to bind specific substates	NSCLC	96
HNRNPC	miR-21	Promote miRNA biogenesis	m6A allow DGCR8 to bind specific substates	Glibolastoma	97
METTL3	pre-miR-143-3p	Promote miRNA biogenesis	Increase Dicer splicing of pre-miRNA	NSCLC	98
METTL3/METTL14	MALAT1	Control lncRNA structure	m6A as a switch	Promote gene expression	106
METTL3	MALAT1	lncRNA-mediated ceRNA model	Sponge miR-1914-3p	NSCLC	107
METTL3	linc1281	RNA-RNA interaction	N/A	Mouse embryonic stem cells differentiation	108
METTL3	XIST	Promote XIST-mediated gene silencing	N/A	N/A	109
YTHDC1	circNSUN2	Promote cytoplasmic export	Forming circNSUN2/IGF2BP2/HMGA2 protein ternary complex	CRC	114
YTHDF3	circRNA	Promote translation	Cap-independent	N/A	115
YTHDF2	circRNA	Promote degradation	HRSP12-RNase P/MRP endoribonuclease	N/A	116

HCC: Hepatocellular carcinoma; NSCLC: Non-small cell lung cancer; CRC: Colorectal cancer.

**Table 2:** ncRNAs modulate the expressions and functions of m6A modifiers

ncRNAs	m6A modifiers	Mechanisms	Cancer types	References
miR-33a	METTL3	Inhibit METTL3 expression	NSCLC	101
miR-600	METTL3	Inhibit METTL3 expression	NSCLC	102
miRNA let-7g	METTL3	Inhibit METTL3 expression	Breast cancer	103
linc00470	METTL3	Promote METTL3 mediated m6A modification	GC	112
lncRNA GATA3-AS	KIAA1429	Promote KIAA1429 mediated m6A modification	HCC	111
miR-1266	FTO	Inhibit FTO expression	CRC	105
lncRNA GAS5-AS1	ALKBH5	Promote ALKBH5 expression	Cervical cancer	110

NSCLC: Non-small cell lung cancer; HCC: Hepatocellular carcinoma; CRC: Colorectal cancer; GC: Gastric cancer.

## Abbreviations

3'-UTRs: 3'-untranslated regions
ALKBH5: AlkB homolog 5
HNRNP: Heterogeneous nuclear ribonucleoprotein
BC: Bladder cancer
CAFs: Cancer-associated fibroblasts
ceRNA: Competitive endogenous RNA
circNSUN2: circular RNA NOP2/Sun RNA methyltransferase 2
circRNAs: Circular RNAs
CRC: Colorectal cancer
DGCR8: DiGeorge syndrome critical region 8
eIF3: eukaryotic translation initiation factor 3
EMT: Epithelial-mesenchymal transition
ERCC1: excision repair cross-complementation group 1
EpCAM: epithelial cell adhesion molecule
FAM225A: Family with sequence similarity 225 member A
FOXM1: Forkhead box M1
GAS5: Growth arrest special 5
FTO: Fat mass and obesity-associated protein
GAS5-AS1: the antisense RNA of GAS5
GATA3-AS: Antisense strand of the GATA binding protein 3 gene
GC: Gastric cancer
GSCs: Glioblastoma stem-like cells
GSK3β: Glycogen synthase kinase 3β
HBXIP: Hepatitis B X-interacting protein
HCC: Hepatocellular carcinoma
HIF-1α: Hypoxia-inducible factor 1-alpha
HMGA2: High mobility group AT-hook 2
lncRNAs: Long noncoding RNAs
m6A: N6-methyladenosine
MALAT1: Metastasis-associated lung adenocarcinoma transcript
METTL14: Methyltransferase-like 14
METTL3: Methyltransferase-like 3
miRNAs: MicroRNAs
mRNAs: Messenger RNAs
ncRNAs: Noncoding RNAs
NEAT1: Nuclear paraspeckle assembly transcript 1
NKAP: NF-κB-associated protein
RISC: RNA-induced silencing complex
NSCLC: Non-small cell lung cancer
Oct4: Octamer-binding transcription factor 4
PDAC: Pancreatic ductal adenocarcinoma
pre-miRNAs: Precursor miRNAs
pri-miRNAs: Primary miRNAs
PTEN: Phosphatase and tensin homolog
RBM15/15B: RNA-binding motif protein 15/15B
RBM15: RNA-binding motif protein 15
SNHG20: Small nucleolar RNA host gene 20
WTAP: Wilm's tumor-associated protein
XIST: X-inactive specific transcript
YAP: Yes associated protein
YTH: YT521-B homology
YTHDC1: YTH domain-containing proteins 1
